# Nonpharmaceutical interventions in Turkey and worldwide during COVID-19 pandemic

**DOI:** 10.3906/sag-2106-210

**Published:** 2021-12-17

**Authors:** Mustafa Necmi İLHAN, Hakan TÜZÜN, Rahmi KILIÇ, Nuran YILDIRIM

**Affiliations:** 1 Department of Public Health, Faculty of Medicine, Gazi University, Ankara Turkey; 2 Member of COVID-19 Community Sciences Advisory Board of Ministry of Health; 3 Department of Otolaryngology, Ankara Education and Research Hospital, Ankara Turkey; 4 Department of Medical History and Ethics, Faculty of Medicine, Bezmialem Vakıf University İstanbul

**Keywords:** Nonpharmaceutical interventions, COVID-19, pandemic, public health, Turkey

## Abstract

Nonpharmaceutical interventions (NPIs) are actions apart from getting vaccinated and medications, in order to promote deceleration of the spread of illness among people and communities during pandemic. In this article, we aim to examine NPIs applied in Turkey and worldwide due to the COVID-19 pandemic. Some of the NPIs such as isolation, quarantine, and contact tracing were maintained with updates of the Ministry of Health guidelines in Turkey. Some NPIs including travel and partial or full curfew mobilization restrictions were set in accordance with the various periods by the number of cases. Periods of restrictions at autumn 2021 to summer 2022 are national partial curfews, national extended curfews, local decision-making phase, revised local decision-making phase, partial lockdown, full lockdown and gradual normalization. Mitigation and suppression have been implemented in Turkey with restrictions of varying severity throughout the course of the epidemic. It is seen that the restrictions implemented in Turkey contributed to the flattening of the epidemic curve. Even some countries mainly applied the suppression method, and others applied the mitigation method, in general, it is seen that similar methods were applied with different weights. Examples of different countries demonstrated that NPIs are effective for flattening epidemic curve. NPI have been the main instrument for a year and a half from the beginning of the epidemic to mid-2021 in Turkey as well as worldwide.

## 1. Introduction

The 69th World Health Assembly was held in May 2016. Dr. Margaret Chan, who was the head of the World Health Organization (WHO) at that time, drew attention to the Ebola, MERS coronavirus, Zikavirus outbreaks in the assembly. She mentioned the “*dramatic resurgence of the threat from emerging and reemerging infectious diseases*”, and mentioning her observation as “*the world is not prepared to cope*” World Health Organization (2016). Sixty-ninth World Health Assembly opens in Geneva. [online]. Website https://www.who.int/news/item/23-05-2016-sixty-ninth-world-health-assembly-opens-in-geneva [accessed 25.05.2021].. Soon after this conversation, unfortunately, at the end of 2019, Dr. Margaret Chan was proved right worldwide.

What makes the COVID-19 pandemic different from previous epidemics is not the biological characteristics of the agent nor the spread pattern of the disease. What makes the COVID-19 pandemic different from the previous ones might be that the society affected by the epidemic is a global society with advanced technological tools and high mobility. In this pandemic, vaccine and drug development studies progressed faster than in any previous pandemics. This rapid progress will likely be among the main factors that determine how and when this epidemic will end. However, neither the vaccine nor the medicine is the primary instrument in combating the epidemic. The main tools to combat the epidemic are public health interventions to individuals or the community as in previous outbreaks.

Public health interventions can sometimes be defined as nonpharmaceutical interventions too. Nonpharmaceutical interventions (NPIs) are defined by the “Centers for Disease Control and Prevention” (CDC) as actions, apart from getting vaccinated and medications that people and communities can take to help slowing the spread of illnesses. And according to the CDC, NPI is among the best ways to control pandemics when vaccines are not available yet Centers for Disease Control and Prevention (2016). Nonpharmaceutical Interventions [online]. Website https://www.cdc.gov/nonpharmaceutical-interventions/index.html [accessed 26.05.2021].. However, it should be emphasized that even in conditions that effective drug or vaccine is available, NPIs are fundamental and traditional instrument to tackle the epidemic. 

In this article, NPI applied in Turkey and worldwide due to the COVID-19 pandemic will be examined. 

## 2. Methods of nonpharmaceutical interventions

NPIs for individuals include isolation and quarantine whereas NPIs for the general population include regulations to restrict social mobility. These regulations are grouped roughly under the headings of suppression and mitigation. Besides, obviously interventions for the individual and society are intertwined, so the intervention titles categorized here may contain common examples. For instance, some mitigation politics include case isolation at home and voluntary home quarantine.

Isolation aims to separate the patient with a contagious disease from healthy people. Quarantine aims to separate people who were suspected of being exposed to a contagious disease to see if they become ill Centers for Disease Control and Prevention (2016). Isolation and quarantine [online]. Website https://www.cdc.gov/quarantine/index.html [accessed 26.05.2021].. 

Suppression aims to reduce the reproduction (R) number to less than one and hence to reduce case numbers to low levels. Demand is to eliminate human-to-human transmission via suppression method. Ideally, this method should be maintained throughout the epidemic period until new vaccines are available [1]. Its implementation is not sustainable during the epidemic period.

Mitigation aims to apply NPI methods to reduce the health impact of an epidemic. The aim is not to target interrupting the transmission completely. The mitigation method only aims to reduce the R number, but not below one. The purpose of this method is to slow down the spread of the epidemic [1]. This can prevent excessive increased demand for healthcare services from exceeding the existing healthcare supply during the epidemic. In case of no restriction measures were taken during the epidemic, certainly the demand for healthcare services would exceeds the supply of healthcare Organisation for Economic Co-operation and Development (2021) Flattening the COVID-19 peak: Containment and mitigation policies [online]. Website https://www.oecd.org/coronavirus/policy-responses/flattening-the-covid-19-peak-containment-and-mitigation-policies-e96a4226/ [accessed 26.05.2021].. 

General social distance, widespread testing, case isolation, contact tracing, university closures are frequently used methods in the scope of mitigation strategy. Closure of primary schools is rarely implemented by the mitigation strategy. Other characteristics of the mitigation strategy are as follows. Travel restrictions are only implemented for high-risk regions or countries. Gathering is restricted according to the number of people. Restrictions for public spaces including cafes, restaurant, shopping malls are applied according to the course of the epidemic [2]. 

 “Contact tracing” is defined as quarantine practice for individuals contacted with infected people. Contact tracing is the main public health intervention to find a source of infection. The spread of the epidemic can be limited with complying to contact tracing [3]. Contact tracing is particularly crucial in this pandemic, considering the fact that significant proportion of COVID-19 patients have no symptoms.

There are some additional important restrictions on the suppression strategy according to mitigation. There are travel restrictions, gathering is forbidden and the schools are frequently closed. There are restrictions for public spaces including shopping malls, restaurants. Finally, the suppression strategy generally includes curfew [2]. 

## 3. Nonpharmaceutical interventions against COVID-19 pandemic in Turkey 

Relevant public health laws and regulations have been published on public health and communicable diseases since the beginning of the foundation of Republic of Turkey. As the latest recent development in the notification system of infectious diseases, an early warning and response system was established in 2007 in Turkey for the surveillance of communicable diseases [4].

It is stated in the Ministry of Health’s guide that the COVID-19 epidemic management is carried out within the framework of the “Pandemic Influenza National Preparation Plan” with intersectoral cooperation under the coordination of the Ministry of Health. The impact of the COVID-19 measures taken by central institutions and organizations is increased by the provincial-specific evaluations made by the Provincial Pandemic Committees [5]. 

Various electronic registry applications have been used for contact screening in Turkey such as “Laboratory Information Management System”, “Public Health Management System”, “Contact Tracing and Isolation Tracking System”, and the “Family Medicine Information System”[6]. While some of these are recording systems that were routinely used before the epidemic, some are particularly developed for epidemic management.

Some practices in Turkey, such as isolation, quarantine, and contact tracing, were maintained with minor changes by making technical updates with the guidelines of the Ministry of Health. Whereas social mobilization restrictions such as curfews and travel restrictions were shaped according to an implementation schedule that changes according to the number of cases. Nonpharmaceutical interventions in Turkey will be listed in two separate sections, the period from the beginning of the pandemic to the autumn of 2021 and the period from the autumn of 2021 to the beginning of the summer of 2022.

### 3.1. Nonpharmaceutical interventions from the beginning of the pandemic to autumn 2020

The first COVID-19 cases were seen in Turkey on 11 March 2020. The first part of the restriction measures implemented during the epidemic is the measures taken from the beginning of the epidemic until the autumn of 2020, including the summer of 2020. Some restriction measures implemented in Turkey during these periods were as follows Wikipedia (2021) Türkiye’de COVID-19 pandemisi zaman çizelgesi [online]. Website https://tr.wikipedia.org/wiki/T%C3%BCrkiye%27de_COVID-19_pandemisi_zaman_%C3%A7izelgesi [accessed 27.05.2021].. 

16 March 2020: Education in Turkey was suspended.

20 March 2020: Curfew was declared for those over the age of 65.

3 April 2020: Intercity travel was restricted in 31 provinces.

4 April 2020: Curfew was declared for those under the age of 20.

10 April 2020: Curfew was declared in 31 provinces on weekends.

11 May 2020: The first phase of the normalization calendar was declared. Barbers, shopping malls, marketplaces, restaurants and cafes were opened.

27 May 2020: Hotels and hostels started accepting guests under favorable conditions.

1 June 2020: Concept of “new normalization” was declared. Public entertainment venues, resting places, tea gardens, association clubs, swimming pools and sports halls were opened. Wedding halls were opened on the condition of not exceeding 25% capacity.

1 July 2020: Restrictions were lifted in wedding venues, theaters, and performance centers.

### 3.2. Nonpharmaceutical interventions from autumn 2020 to summer 2021

National partial curfews which was declared after normal period in summer 2020 started on November 18th T.C İçişleri Bakanlığı (2020) Koronavirüs Salgını Yeni Tedbirler, 18.11.2020 [online]. Website https://www.icisleri.gov.tr/koronavirus-salgini-yeni-tedbirler [accessed 08.06.2021].. The first daily number of COVID-19 cases was reported by the Ministry of Health on November 25th T.C. Sağlık Bakanlığı (2021) COVID-19 Bilgilendirme Platformu [online]. Website https://covid19.saglik.gov.tr/ [accessed 09.06.2021].. There were implemented various nonpharmaceutical interventions during the COVID-19 pandemic in Turkey. We have categorized nonpharmaceutical intervention periods according to some characteristics of the restrictions they contain. While naming the periods, if there was a definition made for that period with the circulars of the Ministry of İnternal Affairs, we used that definition. Table shows the periods of restrictions in Turkey since November 18th 2020 T.C İçişleri Bakanlığı (2021) İç İşleri Bakanlığı Duyuruları [online]. Website https://www.icisleri.gov.tr/duyurular [accessed 01.07.2021]..

**Table:** Periods of restrictions in Turkey since November 18th 2020.

Periods of restriction	Implementationdates
National partial curfews	18.11.2020–30.11.2020
National extended curfews	01.12.2020–28.02.2021
Local decision-making phase	01.03.2021–29.03.2021
Revised local decision-making phase	30.03.2021–13.04.2021
Partial lockdown	14.04.2021–28.04.2021
Full lockdown	29.04.2021–16.05.2021
Gradual normalization	17.05.2021–31.05.2021
2th phase of gradual normalization	01.06.2021

Figure shows the number of cases per day after November 25th in Turkey. The data of the Ministry of Health was used as the data source7. A web application containing Turkey data was used in the database creation process [7].

**Figure 1:**
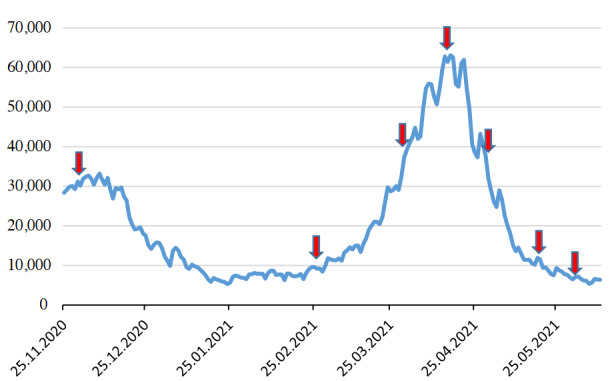
Number of cases per day after the 25th November in Turkey (arrows indicate the onset date of the of implementation of restriction period).

#### 3.2.1. National partial curfews (from 18 November 2020)

A curfew was imposed on weekends except from 10:00 to 20:00. The application started for the first time on November 21st. Eating and drinking places such as restaurants, patisseries, cafes started to work between 10:00 and 20:00, only to provide takeaway or pick-up service. Specific restriction rules were set for age groups. People over the age of 65 were allowed to go out between 10:00 and 13:00, while those under the age of 20 were allowed to go out between 13:00 and 16:00 during the day6. We called this period “national partial curfews”. In general, first period can be considered as the period in which the restriction applications were at the minimal level nationally, except for the last period only (second phase of gradual normalization).

The ministry of health used the terms *patient* and *case* with different definitions when explaining the data about the epidemic. The case of positive PCR tests performed only on people with symptoms was defined as a patient. The case of positive PCR tests performed on all people with or without symptoms was defined as a case. As of November 25th , the number of daily cases began to be announced7 .

#### 3.2.2. National extended curfews (from 1 December 2020)

The scope of curfews has been expanded. A curfew was imposed for the entire weekend, starting at 22:00 on Friday evening. Another curfew was imposed on weekdays starting at 21:00 in the evening T.C İçişleri Bakanlığı (2020) Koronavirüs ile Mücadele Kapsamında - Yeni Kısıtlama ve Tedbirler Genelgeleri, 01.12.2020 [online]. Website https://www.icisleri.gov.tr/koronavirus-ile-mucadele-kapsaminda-sokaga-cikma-kisitlamalari---yeni-kisitlama-ve-tedbirler-genelgeleri [accessed 09.06.2021].. We called this period “national extended curfews”.

#### 3.2.3 . Local decision-making phase (from 1 March 2021)

A *“Local decision-making phase”* was declared from March 1st, 2021. Provinces were categorized as “low, medium, high and very high” according to their risk status. Four different risk classifications were done and named as blue-yellow-orange-red provinces. The decision was to change the classification of the provinces every two weeks according to the current status of the province. Weekend curfews were completely removed in low and medium-risk provinces, while proceeded on Sundays in high and very high-risk provinces. In low and medium-risk provinces, the bans for those over 65 and under 20 were lifted, education begun at all levels of education, and the curfew on the weekend had been lifted. In high and very high-risk provinces, only 8th, 12th grades, primary schools and preschool education institutions were opened. The curfew is not over for those over 65 and under 20, but the curfew had been increased. Going out on Sunday was banned only on weekends. Except for very high-risk provinces, public spaces such as cafes and restaurants started to accept customers again with 50% capacity. The curfew continued throughout Turkey between 21:00–05:00 Wikipedia (2021) Türkiye’de COVID-19 pandemisi 2021[online]. Website https://tr.wikipedia.org/wiki/T%C3%BCrkiye%27de_COVID-19_pandemisi [accessed 17.05.2021]., T.C İçişleri Bakanlığı (2021) 81 İl Valiliğine Lokanta, Restoran, Kafe vb. İşyerleri; Park, Piknik Alanları; Mesire Yerleri ve Giyim Pazarları Genelgesi [online]. Website https://www.icisleri.gov.tr/81-il-valiligine-lokanta-restoran-kafe-vb-isyerleri-park-piknik-alanlari-mesire-yerleri-ve-giyim-pazarlari-genelgesi [accessed 28.05.2021].. 

#### 3.2.4. Revised local decision-making phase (from 30 March 2021)

Curfews on weekdays and on weekends, which had been arranged according to risk groups, were rearranged. The weekend curfew was applied to cover Sundays in the high-risk provinces, and Saturdays and Sundays in very high-risk provinces. Customers were accepted with a 50% capacity limitation in public spaces between 07:00 and 19:00. A maximum of 4 people in the provinces in the low and medium risk groups and 2 in the provinces in the high and very high-risk groups were allowed to sit at the same table at the same time T.C İçişleri Bakanlığı (2021) 81 İl Valiliğine Koronavirüs Tedbirlerinin Gözden Geçirilmesi Genelgesi Gönderildi [online]. Website https://www.icisleri.gov.tr/81-il-valiligine-koronavirus-tedbirlerinin-gozden-gecirilmesi-genelgesi-gonderildi [accessed 27.05.2021].. We called this period “revised local decision-making phase”.

#### 3.2.5. Partial lockdown (from 14 April 2021)

A *“partial lockdown”* was declared from April 14th, 2021. The hours of the curfew on weekdays were updated as 19:00 in the evening and 05:00 in the morning5. Some additional measures were taken due to Ramadan. A weekend curfew was declared in all provinces. Public areas such as restaurants and cafes were closed. Hürriyet gazetesi (2021) Koronavirüs vakaları artınca yeni tedbirler gündeme geldi! [online]. Website https://www.hurriyet.com.tr/gundem/ramazan-yasaklari-41782587 [accessed 28.05.2021].

#### 3.2.6. Full lockdown (from 29 April 2021)

A* “full lockdown”* was declared from April 29th, 2021. Education was suspended at all levels and exams were postponed. It was announced that intercity public transport vehicles will operate at 50% capacity.5^,^ T.C İçişleri Bakanlığı (2021) 81 İl Valiliğine Tam Kapanma Tedbirleri Genelgesi Gönderildi [online]. Website https://www.icisleri.gov.tr/81-il-valiligine-tam-kapanma-tedbirleri-genelgesi-gonderildi [accessed 28.05.2021]. 

#### 3.2.7. Gradual normalization (from 17 May 2021)

A period called “gradual normalization” was declared from May 17th, 2021. The curfew was imposed between 21:00–5:00 on weekdays, and on weekends to cover the whole Saturdays and Sundays and to be completed at 05:00 on Mondays. Public places (such as restaurants, cafeterias, patisseries) were allowed to serve as take-away T.C İçişleri Bakanlığı (2021) Kademeli Normalleşme Tedbirleri Genelgesi [online]. Website https://www.icisleri.gov.tr/kademeli-normallesme-tedbirleri-genelgesi [accessed 28.05.2021].. 

#### 3.2.8. Second phase of gradual normalization (from 1 June 2021)

“Gradual normalization” finished. The second phase of gradual normalization started. A curfew rule was introduced between 22:00 and 05:00 on Mondays and Saturdays, and whole day on Sundays. Food services and drinking places (such as restaurants, cafeterias, and patisseries) were allowed to serve 2 m in all directions between tables and 60 cm between side-by-side chairs provided that all the rules specified in the Epidemic Management and Working Guide of the Ministry of Health were followed. Food services and drinking places operated only between 7:00–24:00 on Sundays and between 21:00–24:00 on other days only as takeaway T.C İçişleri Bakanlığı (2021) Haziran Ayı Normalleşme Tedbirleri Genelgesi, 01.06.2021 [online]. Website https://www.icisleri.gov.tr/haziran-ayi-normallesme-tedbirleri-genelgesi [accessed 01.06.2021]..

## 4. Examples of nonpharmaceutical interventions against COVID-19 pandemic in worldwide

While some countries predominantly applied the suppression method, others predominantly applied the mitigation method. The suppression policy which targets to reduce the R-value below one was implemented by China, Japan, Singapore and Thailand. The mitigation strategy was mostly implemented by European countries (especially England, Italy and France) and the United States [8].

In a study evaluating the curfew and quarantine measures during the second wave of the pandemic in France, it is stated that since September 23rd–25th, social gatherings have been limited in nine metropolises, bars and restaurants have been closed. A curfew was imposed from 21:00 to 06:00 on 17 October, and quarantine was implemented across the country on 30 October. It was reported that 7–10 days after the introduction of these measures, a significant decrease in the incidence of COVID-19 cases and hospitalizations was observed [9]. 

Due to the increase in the number of cases and deaths at the end of February 2020 in Italy, measures such as closure of schools, cancellation of meetings and restriction of travel were introduced. At the end of March, the restrictions increased further. On 22nd of March, all nonessential production, industry and businesses in Italy were closed, providing financial support to the self-employed, healthcare workers, seasonal workers, families, regulations have been introduced. In terms of occupational health and safety, a protocol was signed among the government, unions, and companies to regulate the working environment. At the beginning of May, R_t _fell below one in all regions of the country. After that, the transition to the normal process started. Employees returned to work, funeral ceremonies and home visits were allowed under certain conditions. Restaurants, shops etc. opened for use under certain conditions [10]. 

In the early stages of the pandemic, during the periods when continental European countries such as Italy, Spain and France took the measures which were rejected in the UK. Events where large groups gather, such as sporting events, were not restricted until mid-March 2020. In the UK, it was announced that the herd immunity strategy would be followed before, but later this strategy was abandoned. On March 25th, 2020, the Coronavirus law was enacted. It was stated that the law had a flexible structure that could change according to developments and supports the capacity of public institutions to respond to the epidemic. As of the second half of March, suggestions for restricting social mobilization started to be presented. At the end of March, schools were closed, some flights were banned, and some businesses were banned from opening. In mid-April 2020, elective surgeries were stopped. Schools were scheduled to open in June but were later postponed to September. R_t_ (R transmissibility) was reduced to less than one in the summer of 2020. On the other hand, it rose above one again in October 2020 and later [11].

Unlike Scandinavian countries, which closed their air borders at the beginning of the pandemic, Sweden kept its air borders open and did not apply quarantine to those entering the country. It was stated that the increase in the number of COVID-19 cases and the number of COVID-19-related deaths in the country where the necessary measures to ensure social distance were not mandatory, was higher than in the neighbouring Scandinavian countries [12]. 

At the beginning of the pandemic in Russia, quarantine was imposed for those coming from abroad, and camera networks with facial recognition systems were used to monitor this process in Moscow. In the following periods, although some countries were exempted, PCR testing started to be requested upon entry to the country. Social mobilization restrictions were also applied in various periods. For example, paid leave was applied between March 28th and May 11th, 2020. Schools have been closed as of March 23rd, 2020. On some dates, citizens over the age of 65 and diagnosed with chronic diseases were quarantined at home. After the first restrictions, the transition to normal life started on 12 May 2020. After the peak period in November–December 2020, the number of cases continued to decrease. The decrease continued in the summer months when the restrictions were lifted and normalization started, but the upward trend resumed with September [13].

In order to control the pandemic in Saudi Arabia, measures were taken to ensure social distance, such as closing schools and starting distance education, suspending sports and social activities, working in public and private workplaces. When it was seen that the number of cases continued to increase with these measures, a mandatory curfew was introduced in all cities from 19:00 to 06.00, then the curfews were extended, and even a twenty-four-hour ban was implemented in some cities. It is stated that the public was encouraged to abide by the rules, but fines and imprisonment were imposed on those who violated the curfew. In addition, measures such as suspending Umrah visits and reducing the number of visitors accepted for Hajj were also implemented. By means of all these measures, the pandemic has been brought under control in the country [14].

From the early stages of the pandemic, Latin American countries started to implement measures such as closing borders, reducing mobility during the day, curfews at night, postponing commercial activities and banning intercity travels. It has been reported that the bed occupancy rate decreased in a tertiary hospital in Brazil where COVID-19 patients were followed after the quarantine application [15]. 

In Australia, the number of cases decreased during July thanks to the measures such as mandatory quarantine for returnees, closing bars, entertainment venues, churches and places of worship, and limiting restaurants and cafes to takeaway. After the quarantine practices became more widespread and new measures were taken, the number of new cases decreased to zero in November and it was stated that the epidemic was under control[16]. 

The pandemic started in Thailand in January 2020, and the peak numbers were reached in March 2020, and quarantine was initiated in April 2020. The quarantine was successful by wearing masks, ensuring social distancing and imposing a curfew from 10 am to 4 am, thus reducing the number of cases and mortality [17]. 

Experiencing a sharp increase in COVID-19 cases early in the pandemic, South Korea rapidly controlled transmission while implementing less stringent national social distancing measures than countries in Europe and the USA. The strategy of South Korea was “test, trace, isolate”. Despite less stringent “lockdown” measures, strong social distancing measures were implemented in high-incidence areas and studies measured a considerable national decrease in movement in late February. Measures implemented in South Korea were contact tracing, strong social distancing, and regional implementations. Testing the capacity was swiftly increased, and protocols were in place to isolate suspected and confirmed cases quickly. It is stated that factors affecting negatively struggling pandemic for other countries may be large population widespread geographically and difficulties related finding, testing, isolating cases [18].

The Asian strategy was implemented as very rapid lockdown to contain the infection and follow-up measures to suppress the virus spread. A complete lockdown was implemented in China and a moderate lockdown was implemented in Japan. The combination of strong suppression with controlled release has been described as “hammer and dance” strategy [19].

Studies involving data from more than one country also show the epidemic prevention effect of NPIs. For instance, a systematic review including observational and modeling studies written on contact tracing, screening, quarantine and isolation shows that basic reproduction number (R0) was reduced from 3.11 to 0.21 thanks to rapid contact tracing. ​According to this study, wide quarantine would prevent 79.27% of deaths and 87.08% of infections [20]. In a study aiming to examine the effect of quarantine on the prevalence and mortality of the COVID-19 pandemic, the data of 27 countries in different continents that applied quarantine in May and June 2020 were examined. After 15 days of quarantine, there was a downward trend in the rate of increase in the number of daily cases and daily deaths. However, it was reported that there was no significant decrease in the prevalence and mortality of the disease when compared to the 15 days before and during the quarantine period [21].

## 5. Conclusion

Due to the lack of sufficient evidence-based data on this subject, an assessment could not be made of how applicable the legally declared restrictions are at the national level. Especially for the “local decision-making phase” and “revised local decision-making phase” periods when the number of cases increased, application deficiencies and application differences may be among the possible factors leading to the current result. 

Another important point about restriction measures is that the measures do not fully cover working people. About 61% (16.4 million) of employment was in the lockdown-free sectors. About 22% (6 million) of employment was in the partially exempt sectors. Only 17% (4.4 million) worked in the sectors covered by the full lockdown DİSK-AR (2021) “Tam kapanma” yok, on milyonlar çalışmaya devam ediyor! [online]. Website http://arastirma.disk.org.tr/?p=5712 [accessed 18.07.2021].. This may have played a role as an important factor hindering the positive effect of the restriction measures.

Mitigation and suppression have been implemented in Turkey with restrictions of varying severity throughout the epidemic. It is seen that the restrictions implemented in Turkey contributed to the flattening of the epidemic curve. In this way, a crisis in which the provision of health services does not meet the demand for health services is prevented.

1. Ferguson N, Laydon D, Gilani GN, Natsuko I, Kylie A et al. Report 9: Impact of non-pharmaceutical interventions (NPIs) to reduce COVID-19 mortality and healthcare demand. 2021. doi: 10.25561/77482

2. Kayı İ, Sakarya S. Policy analysis of suppression and mitigation strategies in the management of an outbreak through the example of COVID-19 pandemic. Infectious Diseases and Clinical Microbiology 2020;2(1):30-41. doi: 10.36519/idcm.2020.0009

3. Contact tracing in the context of COVID-19. In: WHO, editor.: World Health Organization; 2021.

4. Keskinkiliç B, Shaikh I, Tekin A, Ursu P, Mardinoglu A, Mese E.A. A Resilient Health System in Response to Coronavirus Disease 2019: Experiences of Turkey. Frontiers in Public Health. 2020;8:577021. doi: 10.3389/fpubh.2020.577021

5. COVID-19 Temaslı Takibi, Salgın Yönetimi, Evde Hasta İzlemi ve Filyasyon, 4 Mayıs 2021. T.C Sağlık Bakanlığı.

6. Şimşek AÇ, Kara A, Baran-Aksakal FN, Gülüm M, İlter B et al. Contact tracing management of the COVID-19 pandemic. Türk Hijyen ve Deneysel Biyoloji Dergisi. 2020;269. doi: 10.5505/TurkHijyen.2020.80688

The suitability and adequacy of restriction practices have been at the forefront of the discussion topics of the pandemic in all countries of the world. It is suggested that while health policies were developed for the epidemic, evaluations should be made by taking into account the unique conditions of the countries. Planning should also be made according to national conditions and evidence-based data. Mixed models should be applied according to the needs [2].

It is seen that NPIs have been applied in different countries in similar ways but with different intensities. Due to the dynamic course of the epidemic, cultural differences between societies, and differences between health systems, it should be considered natural that there are variations in NPI-related practices between different countries. Curfews have been effective in reducing the number of cases in other countries as well as in Turkey. Experiences of countries show that, rapid contact tracing and local area-specific measures seem to be very effective. However, wide geography and large population play a restrictive role in the effectiveness of measures other than curfews. Of course, the prolongation of the time limits the sustainability of NPI.
